# Genome Sequence of *Staphylococcus aureus* Strain LCT-SA67, a Space Flight Strain with Altered Carbon Source Utilization Properties

**DOI:** 10.1128/genomeA.00095-14

**Published:** 2014-02-20

**Authors:** Junfeng Wang, Yinhu Li, Jun Guo, Xuelin Zhang, Yinghua Guo, De Chang, Tianzhi Li, Guogang Xu, Wenkui Dai, Changting Liu

**Affiliations:** a Nanlou Respiratory Diseases Department, Chinese PLA General Hospital, Beijing, China; b BGI-Shenzhen, Shenzhen, China

## Abstract

An increasing number of studies have confirmed that space flight environments can have a significant effect on a variety of microbial properties. To explore the effect of these environments on *Staphylococcus aureus*, we present the draft genome sequence of an *S. aureus* strain, named LCT-SA67, which was isolated after space flight.

## GENOME ANNOUNCEMENT

A *Staphylococcus aureus* strain originated from the China General Microbiological Culture Collection Center (CGMCC) and was labeled CGMCC 1.2386. To determine the response of *S. aureus* to space flight environments, this strain was loaded onto the Shenzhou VIII spacecraft, which flew in space from 1 to 17 November 2011, for a total of 398 h (1). After the Shenzhou VIII spacecraft landed, an *S. aureus* isolate named LCT-SA67 was selected according to Biolog’s powerful carbon source utilization technology. The strain was sequenced at the Beijing Genomics Institute (BGI) (Shenzhen, China) using a HiSeq 2000 platform (Illumina, San Diego, CA).

Genomic DNA isolated from *S. aureus* LCT-SA67 was used to construct small-fragment (with 350-bp inserts) and large-fragment (with 6,000-bp inserts) randomized sequencing libraries. Using SOAP*denovo* (http://soap.genomics.org.cn/), 310 Mb of filtered high-quality reads from the small fragment library were assembled into 58 contigs, and the contigs were assembled into 5 scaffolds based on 120 Mb of paired-end information from the large fragment library. The draft genome of strain LCT-SA67 was estimated to be approximately 2.8 Mb, with a G+C content of 32.71%. There are 2,599 predicted open reading frames, with a total length of 2,337,468 bp, resulting in a coding intensity of 83.72%. The results of COG annotation analysis demonstrated that more genes were predicted to have roles in amino acid transport and metabolism than in the other 20 functional categories. KEGG annotation determined that there are 344, 289, and 268 genes involved in the pathways of membrane transport, carbohydrate metabolism, and amino acid metabolism, respectively, which are the three main classification categories. The predicted genes of *S. aureus* LCT-SA67 were ascribed to 32 GO functional categories, and we also completed Swiss-Prot annotation. The genome contains 4 rRNA operons and 43 tRNA sequences. In addition, 129 tandem repeats, 93 minisatellite DNA sequences, and 5 microsatellite DNA sequences were identified.

### Nucleotide sequence accession number.

This whole-genome shotgun project of *S. aureus* LCT-SA67 has been deposited at DDBJ/EMBL/GenBank under the accession no. ATKO00000000. The version described in this paper is the first version.

## References

[1] Su L Chang D Liu C 2013 The development of space microbiology in the future: the value and significance of space microbiology research. Future Microbiol. 8:5–8. 10.2217/fmb.12.127 23252487

